# CELT^PLUS^ Fat Increases the Metabolic Activity as Well as the SVF-Yield Significantly When Compared to CELT Fat, Even After Cryopreservation with DMSO

**DOI:** 10.3390/cells14161270

**Published:** 2025-08-17

**Authors:** Tom Schimanski, Lukas Prantl, Andreas Eigenberger, Oliver Felthaus, Rafael Loucas, Kirsten Utpatel, Kerstin Steer

**Affiliations:** 1 Department of Plastic, Hand and Reconstructive Surgery, University Hospital Regensburg, Franz-Josef-Strauss-Allee 11, 93053 Regensburg, Germany; 2 Medical Device Lab, Regensburg Center of Biomedical Engineering (RCBE), Faculty of Mechanical Engineering, Ostbayerische Technische Hochschule Regensburg, Galgenbergstraße 30, 93053 Regensburg, Germany; 3 Institute of Pathology, University of Regensburg, Franz-Josef-Strauss-Allee 11, 93053 Regensburg, Germany

**Keywords:** adipose tissue, stromal vascular fraction, extracellular matrix, cryopreservation, DMSO, CELT, CELT^PLUS^

## Abstract

Lipofilling has far more applications than cosmetic surgery alone. Due to its high content of stromal vascular fraction (SVF) cells, lipoaspirate can also be used to treat wounds, as its cellular components may accelerate wound healing. Using our CELT^PLUS^ protocol, we can increase the number of SVF cells per volume. Unfortunately, some patients require more than one treatment to achieve an optimal outcome, but would unnecessarily suffer from repeated liposuction. Therefore, our objective was to test whether cryopreserving CELT^PLUS^ fat could offer a solution, potentially avoiding the need for repeated liposuction procedures. DMSO was used as a cryoprotective agent for proof-of-principle testing, although other non-toxic cryoprotective agents should be considered in the future. The rest of our freezing protocol is a clinically friendly attempt to facilitate the translation into clinical practice. We tested the cryopreserved tissue using histological evaluation, metabolism measurement, SVF cell yield estimation, PCRs from both whole tissue and from cultured SVF cells, and Oil Red “O” staining. We found that freezing CELT^PLUS^ fat with DMSO yields better results than without cryoprotection in all evaluated methods. Until non-toxic cryoprotective agents are tested on CELT^PLUS^ fat, we do not recommend initiating animal or human testing.

## 1. Introduction

Lipofilling is a popular plastic surgery technique that involves taking fat from one area of the body and using it to increase or restore volume in another area. This technique has many applications in plastic surgery and has become increasingly popular in recent years. One of the most common uses of lipofilling is breast augmentation. Instead of using breast implants, which can sometimes feel unnatural and bear the risk of foreign body reaction, lipofilling allows surgeons to use the patient’s own fat to enhance the size and shape of the breasts [1,2,3]. This technique can also be used for breast reconstruction after mastectomy. Lipofilling is also commonly used in facial rejuvenation [4]. As we age, our face loses volume, which can lead to sagging and wrinkles. Lipofilling can be used to restore this lost volume, giving the face a more youthful appearance. This technique can also be used to enhance the cheeks, lips, and chin, or to correct asymmetries and deformities caused by injury or congenital conditions [5]. Lipofilling has also shown promising results in scar revision. By injecting fat underneath scar tissue, lipofilling can help smooth the surface of the skin, promote wound healing, and reduce the appearance of scars [6].

These properties are believed to be mediated by adipose tissue-derived stem cells (ADSCs), endothelial precursor cells, pericytes, and fibroblasts in the lipoaspirate, which are part of the stromal vascular fraction (SVF) [7,8]. These stem cells produce growth factors, such as VEGF or PDGF, which promote revascularization of the wound bed and proliferation of connective tissue [9,10]. We have developed a method of lipoaspirate processing that approximately doubles the SVF concentration per volume and thereby also increases the number of ADSCs: the Cell Enriched Lipotransfere (CELT) protocol [4,9,11,12,13]. Depending on the exact protocol, lipoaspirate processed according to the CELT protocol results in CELT fat (Cleaned and Enriched Lipid Tissue) or CELT^PLUS^ fat (Cleaned and Enriched Lipid Tissue—Purified Long-lasting Ultra-concentrated Supergraft), which are also less susceptible to shear forces, making it ideal for lipofilling in mechanically demanding areas, such as the face, the soles of the feet, or generally near joints [9,12]. Another application for CELT and CELT^PLUS^ fat could be the treatment of chronic wounds, where the use of lipoaspirate has already shown beneficial effects [14,15,16]. Since SVF cells are largely responsible for the observed improvements in wound healing, their increased concentration in CELT and CELT^PLUS^ fat could enhance these therapeutic effects [17,18,19]. Other research groups have already demonstrated beneficial effects of processed lipoaspirate—referred to as SVF-gel in their studies—on wound healing.

Repeated treatments may improve the outcome but involve all the risks and inconveniences associated with undergoing multiple liposuctions [20,21]. Especially when treating patients with impaired wound healing, it is essential to limit the number of sessions to avoid creating more wounds through liposuction, which have the potential to develop into chronic wounds themselves, creating a vicious circle of causing wounds to heal them. One method to avoid multiple liposuctions would be to harvest more lipoaspirate than needed for the first lipofilling and cryopreserve the unused rest for future applications. Since the main goal of lipoaspirate cryopreservation is to maintain SVF viability and yield, it should be possible to cryopreserve CELT and CELT^PLUS^ fat as well, since the main difference is the increased SVF-yield and the denser extracellular matrix. To our knowledge, no comparison of cryopreservation outcomes between differently processed samples using cryoprotective agents (CPAs) has been reported. However, we are aware of two recent studies that investigated the cryopreservation of SVF-gel at −20 °C without the use of additional CPAs: one by Tao, Zhao et al., 2023 [22] and the other by Feng, Hu et al., 2019 [23]. In our opinion, those were well-conducted studies that reported interesting findings. However, Hua, Wei et al., 2023 [24] published an open letter in response to Tao, Zhao et al., 2023 [22], raising two concerns regarding their methodology. The first concern was that −20 °C is not cold enough for long-term cryopreservation, and the second was that the absence of CPAs in cryopreservation does not guarantee the best possible post-thaw outcome. Several studies have shown that the use of CPAs shows superior results compared to dry freezing of lipoaspirate [25,26,27,28,29]. Furthermore, the literature findings suggest a cryopreservation temperature of −80 °C or liquid nitrogen vapor phase [25,27,30,31]. Hua and Wei [24] concluded that cryopreserved SVF-gel lacks clinical relevance. We sincerely think that, with further improvements to the cryopreservation process, we can justify the usage of cryopreserved CELT^PLUS^ fat in clinical usage, as, for example, wound healing improvement, because the results of Tao, Zhao et al. [22] and Feng, Hu et al. [23] showed promising results.

However, it is important to note that cryopreservation of lipoaspirate has limitations. The viability of the fat cells may be reduced during the cryopreservation process, potentially affecting the success of the lipofilling procedure [25,26,30]. A proper freezing and thawing protocol that reduces cell death at these critical stages was invented by Pu et al., 2004, using a programmable methanol freezing bath and a liquid nitrogen freezing tank [28]. However, we will be using a protocol that is compatible with clinical use to facilitate the transition to clinical practice. We aim to determine whether replacing a programmable methanol freezing bath and a liquid nitrogen freezing tank with a conventional Mr. Frosty™ freezing container and a −80 °C freezer will yield acceptable results in our in vitro study. We will utilize DMSO as a CPA, as its usage is well documented and established. However, this will serve as a proof of principle before other CPAs are tested.

In this study we aim to validate the CELT protocol for additional purposes. We will compare the outcome of cryopreservation of CELT fat and CELT^PLUS^ fat with and without DMSO as a CPA.

## 2. Material and Methods

### 2.1. Harvesting and Preparation of Lipoaspirates

**Figure 1:**
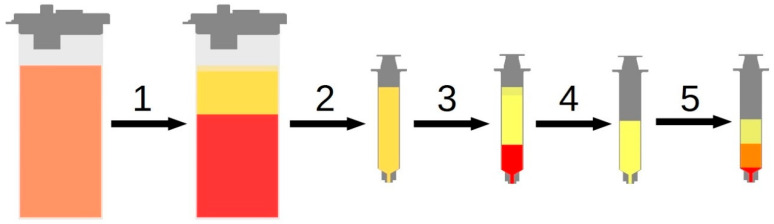
Visualization of our standardized protocol to produce CELT^PLUS^ fat from lipoaspirate: Lipoaspirate is allowed to sediment (1) and is subsequently transferred to a syringe (2). After centrifugation (3) the aqueous phase and the lipid phase are discarded (4), and the CELT fat is subjected to shear forces by intersyringe processing. After a second centrifugation (5) the lipids released by disrupted mature adipocytes are discarded.

Tests were conducted on lipoaspirate obtained from 14 patients (10 female and 4 male) with a mean age of 48.8 ± 12.7 years and a mean BMI of 31.5 ± 6.5 kg/m^2^. All patients gave written consent to the use of their tissues. This study was approved by the Ethics Committee of the University Hospital of Regensburg (08/117). The liposuction was performed as previously described in more detail [13]. Briefly, a tumescent solution containing 0.9% (*w*/*v*) NaCl mixed with epinephrine in a ratio of 1:200,000 is injected into the harvesting site with a 2.5 mm injection cannula and is allowed to incubate for about 15 min. Epinephrine, in such concentrations, is a well-documented and established pharmaceutical agent, with a thirty-year history of use in the field of liposuction to reduce blood loss [32,33]. For harvesting of the lipoaspirate, we use a 3.8 mm cannula (Human Med AG, Schwerin, Germany). Our liposuction device of choice (Body-Jet^®^, Human Med AG, Schwerin, Germany) is a waterjet-assisted liposuction system with constant negative pressure of less than 0.5 mbar. The aspirates were harvested at the Caritas St. Josef Hospital, Regensburg, and were transferred to the University Hospital, Regensburg. Following sedimentation, all collected aspirates were subjected to the respective treatments. For CELT^PLUS^ processing, the lipoaspirate was treated as described earlier in Prantl et al., 2020 [9], as illustrated in Figure 1. Briefly, the lipoaspirate is allowed to sediment for ten minutes to achieve a separation of the adipose tissue from the tumescence solution. Sedimented lipoaspirate is transferred to a 20 mL luer-lock syringe (Becton Dickinson S.A., Madrid, Spain) and centrifuged for 2 min at 1600 rcf (Rotina 380R; Andreas Hettich GmbH & Co. KG, Tuttlingen, Germany), resulting in a big aqueous phase, the tissue phase, and a small lipid phase. The supernatant lipid layer and infernatant tumescence layer are disposed to receive CELT fat. For shear-force intersyringe processing, the syringe is connected to a second 20 mL luer-lock syringe via a three-way stopcock, with an inner diameter of 1.2 mm (B. Braun Melsungen AG; Melsungen, Germany). Now, we vigorously pressed the CELT fat back and forth 10 times for homogenization. We performed this to enrich the fraction of stromal vascular cells in the remaining tissue that are less susceptible to sheer forces [9,12]. The subsequent second centrifugation for 2 min at 1600 rcf results in a big lipid phase from disrupted adipocytes, which is also discarded. The remaining tissue is greatly reduced in volume but has a higher ECM and ADSC concentration. In the following we will refer to the processed tissue after Step 5 as “CELT^PLUS^ fat” and to the unprocessed but centrifuged lipoaspirate after Step 4 as “CELT fat”.

### 2.2. Preparation of CPA Solutions

Dimethyl sulfoxide (Sigma Aldrich, St. Louis, MO, USA) (DMSO) in a concentration of 20% (*v*/*v*, DMSO, α-MEM, and FCS in a 2:4:4 ratio) was used as a CPA. After mixing 1:1 with CELT fat or CELT^PLUS^ fat, we received the final concentrations for the CPA (10% *v*/*v*). Regarding cryopreservation, both the CELT^PLUS^ fat and the CELT fat were treated with one of the following protocols:

Treatment 1: Fresh unfrozen control (control) right after processing;

Treatment 2: Frozen without a CPA (*w*/*o*) for 24 h at −80 °C;

Treatment 3: Frozen mixed with the same volume of CPA (DMSO) for 24 h at −80 °C.

The concentration of a final 10% DMSO was chosen from the literature for the cryopreservation of SVF derived from fat tissue [34,35,36].

### 2.3. Freezing and Thawing Protocol

Control samples were subjected to evaluation immediately. Samples with (DMSO) or without (*w*/*o*) a CPA were put into the −80 °C freezer for 24 h in freezing containers (Nalgene ™ Cryo 1 °C Freezing Container, Thermo Fisher, New York, NY, USA), which ensure a constant freezing rate of 1–2 °C/min. This slow cooling rate reduces artificially induced ice formation (30). All samples were thawed 24 h after freezing using a water bath (Memmert GmbH + Co. KG, Schwabach, Germany) at 37 °C for 3.5 min. This allows sufficient time for the samples to thaw thoroughly. This protocol is a simplified version of the well-documented and experimentally approved ones from Cui et al. [25] and Pu et al. [28], as described earlier in the introduction.

### 2.4. Histological Evaluation

CELT and CELT^PLUS^ samples were fixed in a neutral buffered formalin and embedded in paraffin. The paraffin block was sectioned into 4 µm thick slices. The histological slides were deparaffinized with xylene and rehydrated through a graded alcohol series. After hematoxylin/eosin staining, the slides were digitized using a P1000 slide digitalization system (3D Histech Kft., Budapest, Hungary). The MAs and SVFCs were counted.

### 2.5. Vitality Assay

For vitality determination, 50 µL CELT/CELT^PLUS^ aliquots were incubated with resazurin (Sigma Aldrich, St. Louis, MO, USA) in quadruplicates. The blue resazurin is a cell-permeable redox indicator that can be reduced to the pink resorufin with, in this case, NADH from living, metabolically active cells. This change in fluorescence intensity can be measured with a 560 nm excitation and 590 nm emission filter set. Each sample was filled up with 45 µL of 0.7 M resazurin and 0.405 mL of α-MEM + FCS to bring the combined volume of every sample to 0.500 mL in total. Immediately after mixing we transferred all samples into an incubator with a drive tube rotator at 37 °C, 10 rpm, for 1 h before we centrifuged them once again, now at 525 rcf for 2 min. We then performed a measurement of the fluorescence intensity using 100 µL of each fluid phase from the bottom layer of each cup in single wells of a 96-well plate. The plate reader (VarioScan; Thermo Scientific, Waltham, MA, USA) measures the fluorescence intensity in each well. Since fluorescence intensity is directly proportional to the metabolic activity of each sample, we conclude that cell survival and fluorescence are also linked.

### 2.6. Cell Count

We adjusted our sample to a volume of 0.7 mL of fat to ensure a reasonable amount of SVF cells for the cell culture. All groups were mixed with an isovolumetric amount of 0.2% collagenase type 1 (from Clostridium histolyticum, Sigma Aldrich, St. Louis, MO, USA) and rotated at 10 rpm for an hour at 37 °C. All samples were spun through 250 µm tissue strainers (Thermo Fisher, Pierce^®^ Tissue Strainers, New York, NY, USA) with 500 rcf for 2 min to remove undigested particles. The digestion is then stopped by adding double the volume of α-MEM (Pan Biotech, Aidenbach, Germany) with 10% FCS. The isolated cells were diluted 1:1 (*v*/*v*) with PBS and centrifuged at 200 rcf for 10 min. After resuspending the SVF to a volume of 700 µL each, we took 100 µL suspension and mixed it with 100 µL 0.4% trypan blue (Sigma Aldrich, St. Louis, MO, USA). We then counted the living cells of 10 µL of this mixture in a Neubauer chamber at ×40 magnification.

### 2.7. Cell Culture/Adipogenic Differentiation Potential Cell Media

For the cell culture, α-MEM (Pan Biotech, Aidenbach, Germany) + 10% FCS + 1% penicillin/streptomycin was used. For adipogenic differentiation, this cell culture medium was supplemented with 1 µL/mL of 500 mM IBMX (SERVA, Heidelberg, Germany), 1 µL/mL of 1 mM dexamethasone (Pan Biotech, Aidenbach, Germany), 5.7 µL/mL of 9.5–11.5 mg/mL human insulin (Merck, Darmstadt, Germany), and 2 µL/mL of 100 mM indomethacin (Sigma Aldrich, St. Louis, MO, USA).

The remaining 600 µL of cell suspension from the cell count was cultured in single wells of a 6-well plate (Cellstar™, Greiner Bio-One International GmbH, Gremsmünster, Austria) with a total of 2 mL α-MEM (Pan Biotech, Aidenbach, Germany) + 10% FCS and 1% penicillin/streptomycin, cultivated in a 37 °C 5% carbon dioxide atmosphere. The medium was changed three times a week. After reaching subconfluency, the cells were passaged into a T75 flask (Cellstar™, Greiner Bio-One International GmbH, Gremsmünster, Austria). After the second and final passage of the cells into 6- and 24-well plates (Cellstar™, Greiner Bio-One International GmbH, Gremsmünster, Austria), differentiation assays were conducted. After two weeks of cultivation with an adipogenic differentiation medium, the cells were either harvested by lysis for RNA isolation or were fixed with formalin for subsequent Oil Red O staining.

### 2.8. RNA Isolation, Reverse Transcription, and qPCR

First 150 mg of adipose tissue is mixed with 1 mL of Trizol (TRI Reagent^®^, Sigma Aldrich, St. Louis, MO, USA). We performed this by using two syringes and one three-way stopcock. The homogenisate is then spun at 16,000 rcf for 5 min at 4 °C (Refrigerated Centrifuge Biofuge fresco, Heraeus, Hanau, Germany). After the first centrifugation the red infranatant is transferred into a new 1.5 mL Eppendorf cup to be spun again at 12,000 rcf for 5 min at 4 °C. If there was a lipid layer on top, we discarded it and mixed the infranatant with 200 µL of chloroform. The mixture was shaken for 1 min. We let the mixture incubate at room temperature for 5 min to let the nucleoprotein complex dissociate. We centrifuged once again at 16,000 rcf for 15 min at 4 °C. The clear supernatant is transferred to a fresh Eppendorf cup and mixed with the same volume of 70% ethanol. The whole protocol is a slightly changed version of Zhang et al., 2023 [37].

The RNA from the cell culture was collected using the Qiagen RNeasy Mini kit. The RNA to cDNA transcription was performed using the QuantiTect™ Reverse Transcription Kit from Qiagen (Hilden, Germany), according to the manufacturer’s protocol. The PCRs were performed using the Takyon™ No ROX SYBR^®^ MasterMix blue dTTP (Takyon, Milano, Italy), the primers for GAPDH, PPAR-γ, and C/EBP from Table 1, and cDNA in a three-step real-time PCR.

### 2.9. Oil Red O Staining

Cells that underwent two weeks of adipogenic differentiation were stained with Oil Red O (Sigma Aldrich, St. Louis, MO, USA). After microscopic documentation, cells were destained using 100% isopropanol for quantification. Quantification was conducted by measuring the optical density (VarioScan; Thermo Scientific, Waltham, MA, USA) at 510 nm.

### 2.10. Statistical Analysis

For the resazurin assay and SVF-yield, all results were normalized to the control CELT fat before expressing them as the mean + standard deviation (mean + SD). The Oil Red O staining results, as well as the histological examination, were expressed as the mean + standard deviation (mean + SD). All PCRs were evaluated using the ∆∆Ct method [38,39]. In the present analysis, a paired Student’s *t*-test was employed to compare the data between the different conditions. A *p*-value of <0.05 was considered statistically significant. All groups that were compared using Student’s *t*-test were normally distributed according to the Kolmogorov–Smirnov test. The evaluation of data with limited replication was conducted through descriptive analysis. To assess the effect size, we calculated Cohen’s d.

## 3. Results

### 3.1. Histological Examination

**Figure 2:**
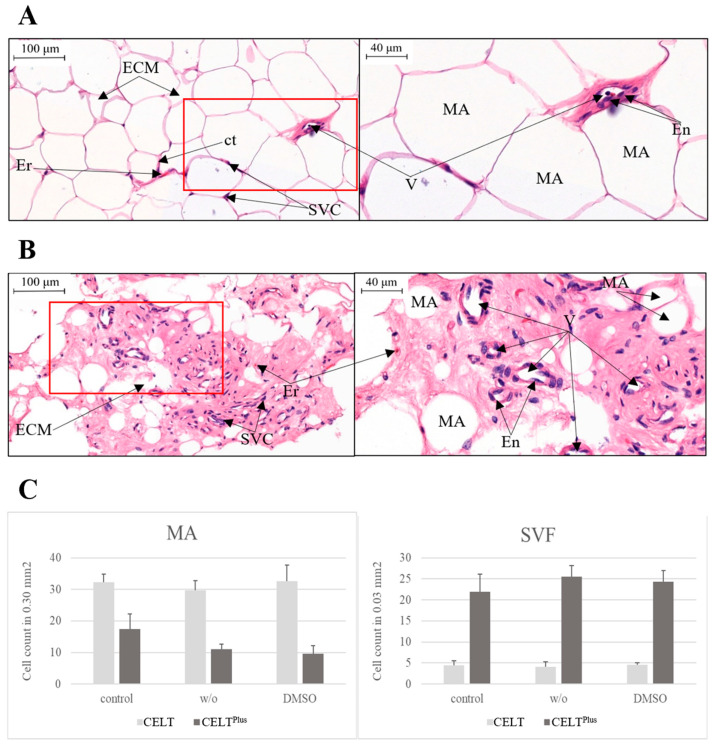
Histological images of control CELT fat (**A**) and CELT^PLUS^ fat (**B**). Mature adipocytes (MA), extracellular matrix (ECM), connective tissue (ct), erythrocytes (Er), vessels (V), endothelial cells (En), and other SVF cells (SVC). Left pictures are in 20-fold magnification and right pictures are clippings (red frame) in 40-fold magnification. (**C**) Showing mean count of mature adipocytes per 0.30 mm^2^ and SVF cells per 0.03 mm^2^.

The CELT^PLUS^ fat group yields more SVF cells than equally treated CELT fat samples and fewer mature adipocytes. Also, the number of intact MAs decreased through the freezing and thawing process while the number of SVF cells increased, as seen in Figure 2.

### 3.2. Vitality Assay and Cell Count

**Figure 3:**
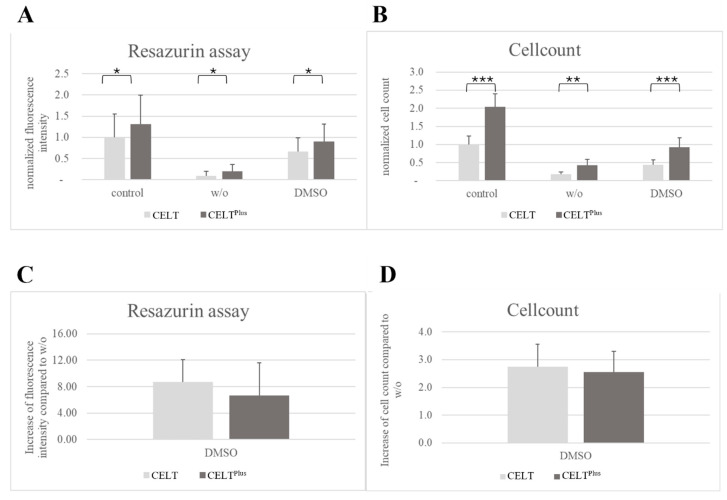
Metabolism of the whole tissue and cell yield of the SVF. (**A**) Resazurin assay normalized to control CELT fat. (**B**) Cell count normalized to control CELT fat. (**C**) The fluorescence intensity from CPA-treated fat divided by the fluorescence intensity from *w*/*o*. (**D**) The cell counts from CPA-treated fat divided by the cell count from *w*/*o*. Please note that the *y*-axis of (**A**–**D**) is not equally scaled. *: *p* ≤ 0.05; **: *p* ≤ 0.01; ***: *p* ≤ 0.001.

The CELT^PLUS^ fresh control samples, on average, had 1.31 ± 0.68 times higher fluorescence intensity than the CELT fat, as seen in Figure 3A. Their not normalized mean values were 686.65 ± 358.22 vs. 524.81 ± 287.29. However, for the frozen samples, the highest values came from DMSO CELT^PLUS^ fat (0.90 ± 0.42 times the value of the control CELT fat group) with significant (*p* < 0.05) differences to all CELT fat samples except control CELT fat, with *p* = 0.34. Cohen’s d comparing the control CELT vs. the control CELT^PLUS^ fat and DMSO CELT vs. DMSO CELT^PLUS^ fat indicates high differences with values of 1.1 and 1.2. The results were normalized to their corresponding control CELT fat absorbance for better comparison. The non-normalized values of CELT DMSO, CELT *w*/*o*, CELT^PLUS^ DMSO, and CELT^PLUS^ *w*/*o* were 350.83 ± 169.15, 49.09 ± 55.20, 472.14 ± 217.97, and 103.78 ± 85.43, respectively.

The CELT^PLUS^ fat control samples, on average, had a 2.05 ± 0.36 times higher yield of SVF cells than their CELT fat counterpart, as seen in Figure 3B. Their non-normalized mean values were 814,500.00 ± 141,835.29 cells/mL vs. 398,071.43 ± 93,582.91 cells/mL. As in the resazurin assay, the DMSO CELT^PLUS^ treatment came out superior to the other frozen groups (0.93 ± 0.26). With significantly higher yields in comparison to all CELT fat samples (with *p* < 0.05) except the control CELT fat, with *p* = 0.54. The CELT^PLUS^ fat DMSO group had the best results for the cell yield of the frozen groups. Cohen’s d comparing the control CELT vs. the control CELT^PLUS^ fat and DMSO CELT vs. DMSO CELT^PLUS^ fat indicates high differences with values of 4.6 and 2.2. The results were normalized to their corresponding control CELT fat cell yield for better comparison. The non-normalized values for cells/mL of CELT DMSO, CELT *w*/*o*, CELT^PLUS^ DMSO, and CELT^PLUS^ *w*/*o* were 173,500.00 ± 53,946.73, 71,666.67 ± 25,385.91, 368,500.00 ± 105,048.80, and 167,777.78 ± 67,827.85, respectively.

As demonstrated in Figure 3C, the differences in the optical density measurements between the DMSO and *w*/*o* groups can be displayed by dividing the optical density of the DMSO groups by that of the corresponding *w*/*o* group. This coefficient is higher for CELT than for CELT^PLUS^ fat, with 8.7 times ± 3.4 (6.7 times ± 5.0 for CELT^PLUS^ fat) higher optical density for CELT. Because of the high standard deviation, no statistically significant differences were found.

As demonstrated in Figure 3D, the differences in the cell count between the DMSO and *w*/*o* groups can be displayed by dividing the cell count of the DMSO groups by that of the corresponding *w*/*o* group. This coefficient is higher for CELT than for CELT^PLUS^ fat, with a 2.7 times ± 0.8 (2.6 times ± 0.8 for CELT^PLUS^ fat) higher normalized cell count for CELT. Because of the high standard deviation, no statistically significant differences occurred.

### 3.3. PCR from Whole Tissue and Cell Culture

**Figure 4:**
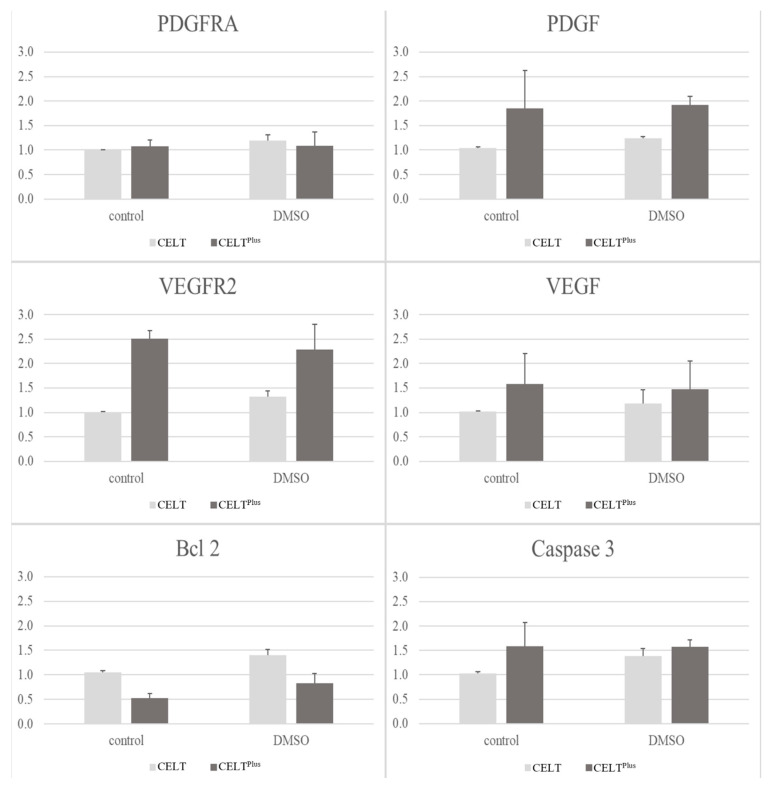
PCRs from whole tissue from control fat, as well as DMSO cryopreserved fat. Important genes for graft retention and regeneration potential (PDGFRA, PDGF, VEGFR2, and VEGF), as well as for apoptosis (Bcl 2 and Caspase 3).

Despite the repeated attempts to collect RNA from the *w*/*o* samples, the quantity obtained was insufficient for the PCR for both whole tissue and the cell culture. Nevertheless, as seen in Figure 4, a comparison of the control and DMSO samples reveals that PDGF and VEGFR2 exhibit the highest increase from CELT to CELT^PLUS^, while for VEGF, only a comparatively moderate increase was measured. The apoptosis marker Bcl 2 demonstrated a decrease in expression, while Caspase 3 exhibited an increase in expression when comparing CELT to CELT^PLUS^ for both control and DMSO samples. A comparison of the control group with the DMSO group reveals no major alterations in PDGFRA, VEGF, and VEGFR2. The expression levels of the apoptotic markers Bcl 2 and Caspase 3 exhibited an increase, except for Caspase 3 in CELT^PLUS^ fat. The expression of PDGFRA was found to be relatively stable across all groups.

**Figure 5:**
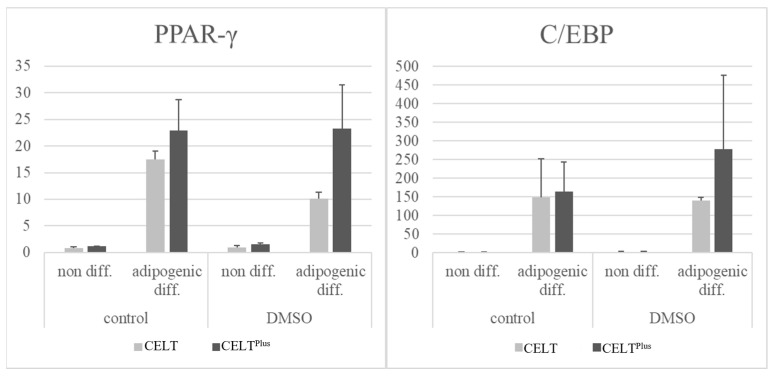
PCRs from cell culture from control fat, as well as DMSO cryopreserved fat. Relative gene expression of marker genes for adipogenic differentiation (PPAR-γ and C/EBP) is shown.

Upregulation of adipogenic marker genes PPARγ and CEB/P was retained after cryopreservation. However, whereas in CELT, upregulation was decreased (PPARγ) or consistent (CEB/P), in CELT^PLUS^, the upregulation was consistent for PPARγ and increased for the CEB/P group, as seen in Figure 5.

### 3.4. Oil Red O Staining

**Figure 6:**
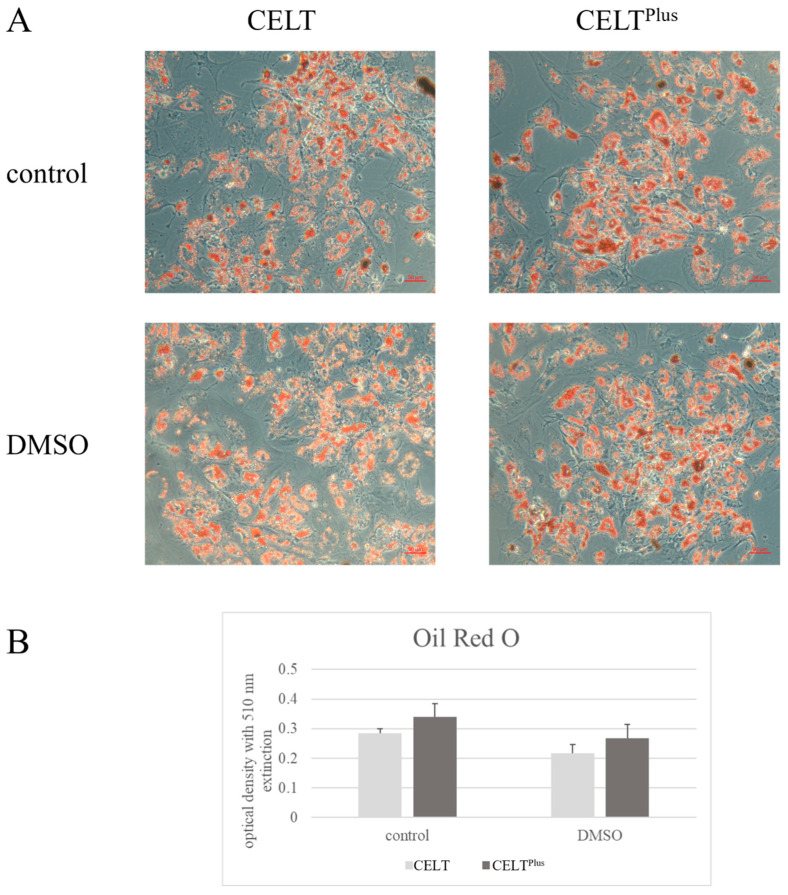
Oil Red O staining after two weeks of differentiation. (**A**) All groups right after staining. The pictures were taken from spots of high density of differentiated cell conglomerates. The red bar in the bottom right corner equals 50 nm. (**B**) The optical density was determined by first destaining the cell cultures with 100% isopropanol and subsequently measuring the optical density of the isopropanol.

The absorbance in the control group is higher than in the corresponding DMSO groups, and the CELT group yields less absorbance than its corresponding CELT^PLUS^ fat group, as seen in Figure 6.

## 4. Discussion

There are many protocols for the cryopreservation of adipose tissue in the form of lipoaspirate [25,26,27,29]. The main novelty of this study is the use of CELT^PLUS^ fat for cryopreservation in comparison to CELT fat. The histological slides in Figure 2 show that the microscopic appearance of the two tissues varies greatly, as do their properties before and after freezing/thawing. It showed that after cryopreservation, the preparation of the samples was of comparable quality, that the composition of the samples was similar, and that potential architectural changes could be described. There is no possibility to determine dead SVF cells in HE staining, while damaged MAs could be detected through a fractured cell membrane. It became clear that post-thawed CELT^PLUS^ fat had fewer architecturally intact MAs than before freezing. This could be because of the two MAs' destructive effects, one being CELT^PLUS^ preparation and the other freeze–thawing. Therefore, the SVF count was slightly higher, as seen in the graphs of Figure 2, probably because the damaged MAs occupy less space due to rupture, and therefore, the SVFCs move together in a tighter space. However, since the integrity of MAs is not that important for clinical use, this finding is bearable. In addition, more vessels are found in CELT^PLUS^, which could potentially lead to higher retention rates. Anyway, the histological appearance shows that the SVFCs can be increased per volume.

Feng et al., 2019 [23] investigated the properties of what they called “cryo-gel”, which is essentially the same as cryopreserved CELT^PLUS^ fat. They tested a mouse model of ischemic wound healing treated with frozen lipoaspirate and cryo-gel. They found accelerated wound healing when cryo-gel was used, with higher retention rates and fewer oil cysts [23]. Due to the absence of CPAs and cryopreservation at −20 °C, they found that normal cryopreserved lipoaspirate did not provide a better wound healing outcome compared to no treatment. This could be explained by our findings that the mean metabolic activity and mean cell count are higher for CELT^PLUS^ fat without any CPA than for CELT fat without any CPA; see Figure 3A,B. Therefore, in Feng et al., 2019 [23], cryo-gel may have been able to deliver a positive effect when aspirate did not.

The increase in cell yield was greater than the increase in metabolism when comparing CELT^PLUS^ fat to CELT fat, with both being significant and with high values for Cohen’s d in the control CELT vs. the control CELT^PLUS^ fat and DMSO CELT vs. DMSO CELT^PLUS^. A 1.31 ± 0.68-fold increase in metabolism and a 2.05 ± 0.36-fold increase in cell yield were recorded when comparing DMSO CELT^PLUS^ fat with DMSO CELT fat. However, Figure 3C,D show that the effect of the CPA on the cell count and the metabolic activity is even stronger for CELT and CELT^PLUS^ fat compared to non-CPA-treated fat (*w*/*o*). This also explains why we were unable to establish stable cell cultures from non-CPA-treated fat. It is also noteworthy that the increase in these properties is higher for CELT than for CELT^PLUS^ fat. Two explanations could be the better survival of CELT^PLUS^ fat in the absence of a CPA due to a protective effect of the thicker extracellular matrix, which becomes less impactful when using a CPA, or a minor cryoprotective effect of a CPA for CELT^PLUS^ fat since the optimum DMSO concentration for CELT^PLUS^ fat could be different from that for CELT fat. Nonetheless, the freezing of CELT fat alone, and the subsequent processing of the thawed product as CELT^PLUS^ fat, is not a viable proposition. The reason for this is that CELT fat would require a substantially greater volume of storage space than CELT^PLUS^ fat. This, in turn, would present a significant challenge in terms of its clinical application. Consequently, the most effective concentration of cryoprotective agents for CELT^PLUS^ fat should be determined to optimize the process.

Since living cells can change their gene expression in a short time to adapt to new circumstances, the method of whole tissue PCR offers many possibilities in the field of lipoaspirate research. Cultured cells are still the method of choice for assessing gene expression. However, for some questions, it is more interesting to spare the cells the time to adapt to cell culture conditions and to look at the gene expression as it is in the lipoaspirate at a certain moment after a specific effect of interest. In our example, the effect of interest is cryopreservation. Whole tissue PCR showed that important parameters of lipograft retention, such as VEGF, VEGFR2, and PDGF, were expressed at higher levels in the CELT^PLUS^ fat than in CELT fat, even after cryopreservation; see Figure 4. These growth factors and the growth factor receptor support the migration, proliferation, and overall survival of the lipograft [40,41,42]. The expression of apoptosis markers indicates a slightly higher rate of apoptosis in CELT^PLUS^ fat, which is most likely due to the mechanical preparation. To further validate these findings regarding the apoptosis, flow cytometry with annexin V and propidium iodide staining should be performed. Nonetheless, given the observation that the living SVF cells and metabolic activity exhibited a significant increase in CELT^PLUS^, this finding only constitutes a minor downside of the protocol. Surprisingly, the cryopreservation process did not appear to increase the rate of apoptosis; in fact, it appeared to decrease it slightly. This phenomenon may be attributable to the fact that cryopreservation predominantly results in necrosis rather than apoptosis. However, further research is necessary to elucidate this observation. Of course, these shifted gene expressions are a manifestation of different ratios of SVFCs to MAs, as seen in the histological examination, since we have already shown that the CELT^PLUS^ protocol does not manipulate the pre-existing cells in their secretom [9]. These findings suggest that CELT^PLUS^ fat keeps its favorable wound healing properties after cryopreservation.

The reconstructive potential of SVF cells is promising [7,43,44,45]. Even after cryopreservation they can still differentiate into adipogenic cell lines [45,46,47,48]. This makes it a target for enrichment in lipografts for various scenarios, such as the treatment of wound healing disorders, where the containment of the SVF has beneficial effects in reducing inflammation and restoring lost tissue volume [6,23,47,49,50]. The semiquantitative evaluation in Figure 5 shows the increase in adipogenic-specific RNA markers, C/EBP, and PPAR-γ, which indicates a successful adipogenic differentiation in vitro, which is preserved even after cryopreservation. PPAR-γ from the DMSO-treated CELT group seems decreased, and C/EBP from the DMSO-treated CELT^PLUS^ fat group seems increased. The quantitative evaluation with Oil Red “O” shows better results in terms of differentiation for CELT^PLUS^ fat than for CELT fat and better results for the control group compared to the DMSO-treated group, as seen in Figure 6.

Because of the short period of time that the samples are frozen, it is not crucial for a successful cryopreservation to freeze in a vapor nitrogen phase [31]. To validate the damage caused by cryopreservation, the freezing and thawing process is the most important part [25]. Also, some protocols from other workgroups that focus on long-term preservation of lipoaspirate transfer their samples from −80 °C to vapor nitrogen after 24 h, at which time we start our thawing [27]. Shu et al., 2015 even recommended storing at −80 °C if the storage time falls below one year [30].

Metabolic activity and cell yield were increased when comparing CPA-treated CELT^PLUS^ fat to non-CPA-treated CELT^PLUS^ fat, and adipogenic differentiation, RNA expression, and histological features were only slightly decreased when comparing cryopreserved CELT^PLUS^ fat to the control CELT^PLUS^ fat. Bearing all this in mind, the clinical potential of cryopreserved CELT^PLUS^ fat does seem very promising. We suggest testing optimal concentrations of non-toxic CPAs for cryopreservation of CELT^PLUS^ fat since this would be necessary for clinical trials. It is also crucial to start in vivo testing of CPA-treated CELT^PLUS^ fat to investigate the retention rate and common side effects, as to our knowledge, this has not been performed to date.

As demonstrated by Zhang et al. (2022), trehalose and glycerol have the potential to be superior to DMSO in terms of cryopreserving lipoaspirate [26]. Further approaches to determine the optimal concentrations of the non-toxic CPAs should be conducted to optimize the cryopreservation of CELT and, particularly, CELT^PLUS^ derivatives. To the best of our knowledge, this has not been undertaken previously. The high post-thaw metabolic activity and the successful differentiation observed in the assay suggest that the applied DMSO concentration did not induce significant toxicity under the conditions of the assay. A further limitation of the present study is that the freezing time was only 24 h. As previously indicated, the extant literature suggests that the freezing and thawing process exerts the greatest damage to the cells, with storage time having a comparatively lesser effect. The present study was conducted to provide proof of concept that cells in CELT and CELT^PLUS^ fat can survive cryopreservation. Nevertheless, the investigation of extended storage periods should be considered, as this is the current focus of the research. The viability, differentiation capacity, and functional recovery of the material, as well as graft retention and vascularization in in vivo models, are currently being investigated to facilitate the next step in the direction of clinical translation.

## 5. Conclusions

Cryopreservation of CELT^PLUS^ fat is possible, and we believe it has great potential for wound healing and lipofilling in mechanically demanding areas. The transdifferentiation capacity of frozen fat was slightly limited, but generally at a high level. CELT^PLUS^ fat also contains higher levels of growth factors compared to normal CELT fat, such as VEGF and PDGF, which are responsible for retention after lipofilling. To validate whether the CELT^PLUS^ protocol was successful, a histological examination should look like Figure 2B.

## Figures and Tables

**Table 1:** Primer pairs used for PCR.

	Forward Primer	Reverse Primer
GAPDH	GGGAGCGAGATCCCTCCAAAAT	GGCTGTTGTCATACTTCTCATGG
PPAR-γ	CACGGAGCTGATCCCAAAGT	TATAGGCTGGGCTTCCCCTT
C/EBP	TATAGGCTGGGCTTCCCCTT	AGCTTTCTGGTGTGACTCGG
PDGF	CCCCTGCCCATTCGGAGGAAGAG	TTGGCCACCTTGACGCTGCGGTG
VEGF	AACCAGCAGAAAGAGGAAAGAGG	CCAAAAGCAGGTCACTCACTTTG
Caspase 3	GCAAACCTCAGGGAAACATT	TTTTCAGGTCAACAACAGGTCCA
Bcl2	ATCGCCCTGTGGATGACTGAG	CAGCCAGGAGAAATCAAACAGAGG
VEGEFR2	AATCTCTGGTGGAAGCCACG	TCTGGGGTGGGACATACACA
PDGFRA	GGGCACGCTCTTTACTCCAT	GCTCTGGGAAACTTCTCCTCC

## Data Availability

The original contributions presented in this study are included in the article. Further inquiries can be directed to the corresponding authors.
